# SpO_2_/FiO_2_ ratio: an estimation of PaO_2_/FiO_2_ ratio in invasive mechanically ventilated patients in high altitude areas

**DOI:** 10.4103/mgr.MEDGASRES-D-25-00214

**Published:** 2026-07-23

**Authors:** Hongmei Yang, Yifeng Chen, Wenlu Wang, Chaoyong Zhou, Xiaolin Zhu, Tianming Cun, Chengyang He, Yaowu Chen

**Affiliations:** Department of Highland diseases and Critical Care Medicine, Lijiang People’s Hospital, Lijiang, Yunnan Province, China; Department of Clinical Pharmacy, Lijiang People’s Hospital, Lijiang, Yunnan Province, China

Dear Editor,

Acute respiratory distress syndrome (ARDS) is a critical illness frequently encountered in intensive care units (ICUs), characterized by high morbidity and mortality rates.^1^ Its core pathophysiological change is oxygenation impairment due to diffuse alveolar damage, necessitating reliable indicators for clinical assessment of oxygenation function.^2^ The arterial oxygenation index (arterial partial pressure of oxygen (PaO_2_)/inspired oxygen fraction (FiO_2_) ratio), which reflects the relationship between PaO_2_ and FiO_2_, has been incorporated into the Berlin ARDS diagnostic criteria since 2011. It serves as a cornerstone for classifying disease severity, guiding treatment strategies, and evaluating clinical outcomes in patients with acute respiratory failure.^3^ Trends in the PaO_2_/FiO_2_ ratio not only support clinical decision-making but also play a pivotal role in recent clinical research and trials.

Blood gas analysis requires measuring PaO_2_ and calculating the PaO_2_/FiO_2_ ratio.^4^ However, this procedure has limitations, particularly for ICU patients requiring repeated dynamic monitoring. It is cumbersome, increases risks of infection and bleeding, and imposes significant nursing burdens and healthcare costs. Moreover, in resource-limited settings such as high-altitude regions, frequent arterial blood sampling is practically challenging due to limited medical equipment, technical personnel, and patient conditions. Consequently, exploring non-invasive, reliable, and reproducible alternatives to the PaO_2_/FiO_2_ ratio has become a research priority.

Previous studies have suggested that the ratio of pulse oxygen saturation (SpO_2_) to FiO_2_ (SpO_2_/ FiO_2_ ratio) can be used to assess and categorize the severity of respiratory failure without the need for arterial blood sampling. Early research demonstrated a strong linear correlation between the SpO_2_/FiO_2_ and PaO_2_/FiO_2_ ratios when SpO_2_ is below 97%. Current methods for estimating PaO_2_ from SpO_2_ include simulations of the hemoglobin dissociation curve and linear correlations between SpO_2_/FiO_2_ and PaO_2_/FiO_2_ ratios, primarily compared using Rice and Ellis equations.^5^,^6^,^7^ However, most existing studies are based on data from sea-level or low-altitude populations, and their applicability in special geographic environments, particularly high-altitude regions, lacks systematic evaluation. In high-altitude settings, reduced atmospheric pressure, lower oxygen partial pressure, and chronic hypoxiainduced compensatory mechanisms significantly alter SpO_2_ interpretation and oxygen transport dynamics, potentially leading to biases in PaO_2_/ FiO_2_ ratio estimates derived from conventional algorithms. Furthermore, most prior research focused on spontaneously breathing or noninvasively ventilated patients, while the correlation and agreement between SpO_2_/FiO_2_ and PaO_2_/ FiO_2_ ratios in critically ill hypoxemic patients requiring invasive mechanical ventilation remain underexplored. Our preliminary studies in high-altitude regions showed significant correlations (*r* = 0.75–0.90) between estimated PaO_2_/FiO_2_ ratios (using these equations) and measured values, but this was limited to non-invasively ventilated patients.^8^,^9^

Thus, systematically investigating the correlation and agreement between SpO_2_/FiO_2_ ratios, estimated PaO_2_/FiO_2_ ratios, and directly measured PaO_2_/FiO_2_ ratios in critically ill hypoxemic patients undergoing invasive mechanical ventilation at high altitudes holds critical clinical value. Additionally, evaluating the performance disparities among estimation methods and the accuracy of SpO_2_/ FiO_2_ ratios in identifying and diagnosing severe hypoxemia in this specific population will provide essential insights for oxygenation monitoring and clinical decision-making in high-altitude critical care settings.

Patients receiving invasive mechanical ventilation support in the ICU of Lijiang Municipal People’s Hospital were selected. A combined retrospective-prospective cohort study was conducted at a single center to maximize data collection while maintaining methodological consistency. The study design leveraged the stable clinical environment of our ICU, where protocols, equipment, and personnel remained consistent throughout the study period. Data from hospitalized patients between June 2022 and May 2023 were first collected retrospectively, followed by prospective data collection from June 2023 to April 2024.

Based on Rice equation, SpO_2_/FiO_2_ = 64 + 0.84 × PaO_2_/FiO_2_, the measured SpO_2_ was substituted into the formula to calculate SpO_2_/FiO_2_ ratio, yielding an estimated PaO_2_/FiO_2_ ratio. Using Ellis equation, SpO_2_ was substituted into the formula to calculate PaO_2_, thereby deriving an estimated PaO_2_/FiO_2_ ratio.^5^,^6^


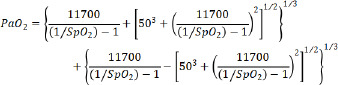


A total of 487 patients were enrolled during the study period. Patients were stratified into two groups based on positive end-expiratory pressure levels: positive end-expiratory pressure < 8 cmH_2_O (1 cmH_2_O = 0.0981 kPa) (*n* = 257) and positive end-expiratory pressure ≥ 8 cmH_2_O (*n* = 230). Baseline characteristics are summarized in **Additional Table 1**. The majority of patients in both groups were female (65.0% *vs*. 67.8%), with median age of 62 (50, 73) years and a median Sequential Organ Failure Assessment score of 6 (5, 8). No statistically significant differences were observed in age, weight, or SpO_2_ between groups (all *P* > 0.05). However, significant differences were noted in PaO_2_, FiO_2_, measured PaO_2_/FiO_2_ ratio, SpO_2_/FiO_2_ ratio, Rice equation-estimated PaO_2_/FiO_2_ ratio, Ellis equation-estimated PaO_2_/FiO_2_ ratio, Sequential Organ Failure Assessment score, duration of mechanical ventilation, and hospital length of stay (all *P* < 0.005).

**Additional Table 1:** Demographic and clinical data

	Total (*n*=487)	PEEP < 8 cmH_2_O (*n*=257)	PEEP ≥ 8 cmH_2_O (*n*=230)	P
Age (yr)	62 (50−73)	62 (50−73)	62 (50−71)	0.125
Sex (female)	323 (66.3)	167 (65)	156 (67.8)	0.507
Height (cm)	168 (162−170)	168 (164−170)	168 (162−170)	< 0.001
Weight (kg)	62 (55−70)	60 (50−70)	63 (58−70)	0.198
FiO_2_ (%)	60 (50−60)	60 (41−60)	60 (60−65)	< 0.000
SpO_2_ (%)	97 (93−99)	97 (93−100)	97 (94−99)	0.154
PaO_2_ (mmHg)	100 (77−148)	100 (76−140)	102 (78−155)	< 0.001
Directly measured PaO_2_/FiO_2_ (mmHg)	186 (131−260)	205 (145−264)	170 (125−257)	< 0.001
Rice equation-estimated PaO_2_/FiO_2_ (mmHg)	120 (106−152)	122 (112−176)	118 (90−122)	< 0.001
Ellis equation-estimated PaO_2_/FiO_2_ (mmHg)	126 (112−174)	138 (125−181)	126 (104−164)	< 0.001
SpO_2_/FiO_2_ ratio	165 (153−192)	167 (158−212)	163 (140−167)	< 0.001
Mechanical ventilation duration (d)	5 (3−8)	5 (3−8)	5 (3−9)	< 0.001
Days of hospital stay	6 (4−11)	6 (4−10)	7 (3−11)	< 0.001
SOFA score	6 (5−8)	6 (5−7)	6 (5−8)	< 0.001

The PEEP threshold of 8 cmH_2_O (1 cmH_2_O = 0.0981 kPa) was selected based on: (1) clinical practice patterns where this level represents a transition point toward more intensive respiratory support; (2) physiological considerations regarding significant alveolar recruitment effects at higher PEEP levels; (3) alignment with published validation studies of oxygenation indices that used similar thresholds to stratify lung injury severity; and (4) consistency with institutional protocols for managing patients with varying degrees of respiratory failure. Continuous variables following a normal distribution are presented as mean ± standard deviation and were analyzed with independent-samples *t*-test; skewed continuous variables are expressed as median (interquartile range) and were analyzed with the Mann−Whitney *U* test. FiO_2_: Fraction of inspired oxygen; PaO_2_: partial pressure of arterial oxygen; PEEP: positive end-expiratory pressure; SOFA: sequential organ failure assessment; SpO_2_: speripheral capillary pulse oximetry oxygen saturation.

The correlation between directly measured PaO_2_/ FiO_2_ ratios, Rice/Ellis equation-estimated PaO_2_/ FiO_2_ ratios, and SpO_2_/FiO_2_ ratios was assessed using intraclass correlation coefficient analysis. A strong correlation was observed (intraclass correlation coefficient = 0.76, *P* < 0.005, 95% confidence interval 0.73–0.79).

In patients with severe hypoxemia (PaO_2_/FiO_2_ ratio ≤ 150), different estimation methods and the SpO_2_/FiO_2_ ratio demonstrated comparable diagnostic accuracy, with areas under the receiver operating characteristic curve (AUC) exceeding 0.70 (**Figure 1** and **Additional Table 2**).

**Figure 1:**
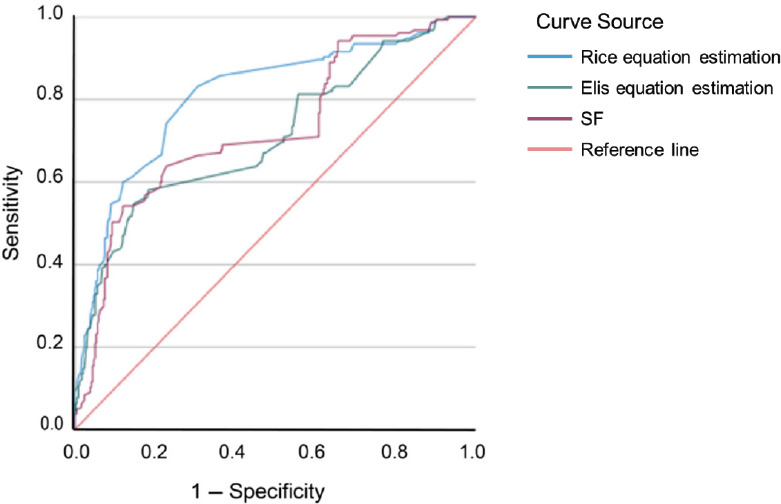
The predictions of diagnostic performance using different estimation methods and SF in patients with severe hypoxemia (SF ≤ 150 mmHg). The performance of each model was evaluated by the area under the curve. ROC: Receiver operating characteristic; SF: the ratio of pulse oximetry (SpO_2_) to inspiratory oxygen partial pressure (FiO_2_).

**Additional Table 2:** Association between Rice equation-estimated PaO_2_/FiO_2_ ratio, Ellis equation-estimated PaO_2_/FiO_2_ ratio, SpO_2_/FiO_2_ ratio, and severe hypoxemia (PaO_2_/FiO_2_ ≤ 150 mmHg)

	AUC	95% CI	Cut-off value	Sensitivity	Specificity	*P*
Rice equation-estimated PaO_2_/FiO_2_ ratio	0.81	0.77-0.85	119.245	0.832	0.693	< 0.001
Ellis equation-estimated PaO_2_/FiO_2_ ratio	0.71	0.66-0.76	117.155	0.548	0.852	< 0.001
SpO_2_/FiO_2_ ratio	0.73	0.68-0.78	156.535	0.542	0.88	< 0.001

The ROC curves demonstrate the predictive performance of three non-invasive methods, Rice equation, Ellis equation, and the SpO_2_/FiO_2_ ratio, for identifying severe hypoxemia, defined as a directly measured PaO_2_/FiO_2_ ratio ≤ 150 mmHg. The analysis included 487 mechanically ventilated patients. The area under the curve for Rice equation-estimated PaO_2_/FiO_2_ ratio (0.81) was higher than that of both Ellis equation (0.71) and the SpO_2_/FiO_2_ ratio (0.73), suggesting superior discriminatory ability. AUC: Area under the ROC curve; CI: confidence interval; ROC: receiver operating characteristic.

To evaluate the practical utility of the SpO_2_/FiO_2_ ratio, we compared the ranges of PaO_2_/FiO_2_ ratios calculated using two methods (Rice and Ellis equations) with the range of directly measured arterial blood gas PaO_2_/FiO_2_ ratios, as summarized in **Additional Table 3**.

**Additional Table 3:** Comparison of PaO_2_/FiO_2_ ratios calculated via different methods with directly measured values

SpO_2_/FiO_2_ ratio	Directly measured PaO_2_/FiO_2_ ratio (mmHg)	Rice equation-estimated PaO_2_/FiO_2_ ratio (mmHg)	Ellis equation-estimated PaO_2_/FiO_2_ ratio (mmHg)
> 300	203−449	288−439	194−496
250−299	205−270	221−273	182−274
200−249	163−238	162−218	111−330
150−199	136−213	102−160	104−264
100−149	75−135	43−101	83−121

Stratification was based on the severity of SpO_2_/FiO_2_ ratio, which was measured directly (line 1: normal/mild; lines 2−5: moderate to severe). Data are presented as interquartile range (25^th^ to 75^th^ percentile).

This study found correlations between PaO_2_/ FiO_2_ ratios estimated via two methods, SpO_2_/FiO_2_ ratios, and directly measured arterial blood gas PaO_2_/FiO_2_ ratios. The non-invasive nature of these indices holds significant clinical value for assessing oxygenation status in invasively ventilated patients, particularly in resource-constrained high-altitude ICUs where arterial blood sampling is challenging. SpO_2_/FiO_2_ ratio emerges as a reliable alternative for dynamic hypoxemia monitoring in such settings. Critically, both estimation methods demonstrated excellent diagnostic performance for severe hypoxemia (PaO_2_/FiO_2_ ratio ≤ 150 mmHg), offering a practical tool for high-altitude critical care.

Prior studies have reported high concordance between non-invasive oxygenation indices and arterial blood gas-derived PaO_2_/FiO_2_ ratio. A previous study reported correlation coefficients ranging from 0.73 to 0.88 using their estimation formula, aligning with our findings (intraclass correlation coefficient > 0.76).^9^ However, the accuracy of non-invasive indices may be influenced by factors such as positive end-expiratory pressure levels, hemoglobin concentration, total bilirubin, determinants of hemoglobin-oxygen affinity (e.g., pH, temperature, carbon dioxide, 2,3-diphosphoglycerate, fetal hemoglobin), and extreme arterial saturation values—variables that often fluctuate in critically ill patients.^4^,^9^,^10^ Against this backdrop, our study reinforces the applicability of SpO_2_/FiO_2_ ratio in high-altitude environments, where low ambient pressure and exaggerated alveolar–arterial oxygen gradients could theoretically compromise accuracy. Even under these conditions, SpO_2_/FiO_2_ ratio reliably reflects oxygenation status in invasively ventilated patients.

Theoretically, hemoglobin dissociation curve-based estimation methods might offer superior precision in hypoxemia diagnosis. Three studies observed enhanced diagnostic performance with Ellis equation for moderate-to-severe hypoxemia.^6^,^7^,^11^ However, our data show comparable diagnostic capabilities between estimation methods, with minimal variation when including patients with SpO_2_ > 97%. This discrepancy may stem from differences in study populations and environmental pressures. Our focus was on severe hypoxemia (not hyperoxia), where SpO_2_ values are less constrained by saturation limits.^10^ Furthermore, as a continuous, dynamic parameter, SpO_2_/FiO_2_ ratio provides unique advantages for real-time oxygenation trend monitoring, offering a simpler and more sensitive reference for ventilator parameter adjustments in ARDS management.

Invasive mechanical ventilation operates within a closed circuit but is not pressure-limited; thus, patients ventilated at high altitudes often exhibit lower PaO_2_ and SpO_2_ values compared to sea-level counterparts. A previous study reported SpO_2_/ FiO_2_ ratios of 214–235 corresponding to arterial PaO_2_/FiO_2_ ratios of 200 in low-altitude residents.^8^ However, no studies have defined an universal SpO_2_/FiO_2_ ratio threshold corresponding to a given PaO_2_/FiO_2_ ratio in mechanically ventilated patients at altitudes exceeding 1500 meters. This gap complicates oxygenation assessment and introduces significant error margins in estimation methods, undermining the accuracy of hypoxemia diagnosis and treatment in high-altitude settings. To address this, we propose an equivalence table linking SpO_2_/FiO_2_ ratio ranges with estimated PaO_2_/FiO_2_ values (via both equations) and directly measured PaO_2_/FiO_2_ ratios. It may aid in diagnosing and following patients with varying degrees of hypoxemia. Notably, Orti et al.^6^ observed comparable diagnostic performance between SaO_2_/FiO_2_ ratios and estimated PaO_2_/FiO_2_ ratios for severe hypoxemia in high-altitude ventilated patients across diverse pathologies, supporting the feasibility of noninvasive oxygenation indices in high-altitude care. Our equivalence table further refines SpO_2_/FiO_2_PaO_2_/FiO_2_ relationships at high altitudes, providing specific quantitative criteria for clinical hypoxemia assessment and foundational data for developing altitude-adjusted ARDS diagnostic criteria.

Certainly, this study has certain limitations. Firstly, single arterial blood gas sampling may introduce random errors, affecting the accuracy of hypoxemia diagnosis. Future studies could enhance data quality by increasing sampling frequency or integrating continuous monitoring technologies. Secondly, retrospective data collection may lead to information bias, which could be mitigated through prospective cohort studies. Lastly, while the sample includes high-altitude populations, it does not cover all altitude gradients. Subsequent research should expand the geographic scope to refine the assessment of oxygenation indices across different elevations.

**Conclusion:** This study demonstrates significant correlations and strong predictive performance between SpO_2_/FiO_2_ ratios and PaO_2_/FiO_2_ ratios in invasively ventilated patients at high altitudes. SpO_2_/FiO_2_ ratio serves as a valid surrogate for PaO_2_/FiO_2_ ratio, offering a simple and reliable method for oxygenation monitoring in high-altitude clinical settings. This finding may enhance the management and outcomes of mechanically ventilated patients in high-altitude regions. The diagnostic efficacy of SpO_2_/FiO_2_ ratios and estimated PaO_2_/FiO_2_ ratios is comparable in severe hypoxemia, though further prospective studies are warranted to explore SpO_2_/FiO_2_ ratio applicability across diverse pathologies and validate its stability in larger, multicenter cohorts.

*This study complied with medical ethics standards and was reviewed and approved by the Medical Ethics Committee of Lijiang Municipal People’s Hospital (approval No. 2022048; dated October 26, 2022). Data collection was performed after obtaining informed consent from the patients or their next of kin.*

*This study was supported by the National Clinical Key Specialty Project of Chinese Emergency Medicine (No. Z155080000004) and Science and Technology Bureau of Lijiang City (No. 2022LJSHFZ016).*

## Additional files

***Additional Table 1:*** *Demographic and clinical data.*

***Additional Table 2:*** *Association between Rice equation-estimated PaO_2_/FiO_2_ ratio, Ellis equation-estimated PaO_2_/FiO_2_ ratio, SpO_2_/FiO_2_ ratio, and severe hypoxemia (PaO_2_/FiO_2_ ≤ 150 mmHg).*

***Additional Table 3:*** *Comparison of PaO_2_/FiO_2_ ratios calculated via different methods with directly measured values.*
